# Utility of the diffusion-weighted imaging for activity evaluation in Crohn’s disease patients underwent magnetic resonance enterography

**DOI:** 10.1186/s12876-015-0235-0

**Published:** 2015-02-05

**Authors:** Feng Qi, Shen Jun, Qiao Yu Qi, Peng Jiang Chen, Geng Xiao Chuan, Zhu Jiong, Xu Jian Rong

**Affiliations:** 1 Department of Radiology, Ren Ji Hospital, School of Medicine, Shanghai Jiao Tong University, Shanghai, China; 2 Shanghai Institute of Digestive Disease, Shanghai Inflammatory Bowel Disease Research Center, Division of Gastroenterology and Hepatology, Ren Ji Hospital, School of Medicine, Shanghai Jiao Tong University, Shanghai, China

## Abstract

**Background:**

Cross-sectional imaging techniques as magnetic resonance enterography (MRE) may offer additional information on transmural inflammation, stricturing and fistulising complications in Crohn’s disease (CD). The purpose of our study was to evaluate the diagnostic accuracy of Magnetic Resonance Imaging (MRI) combined with Diffusion-weighted Imaging (DWI) and MRE for determination of inflammation in small bowel CD.

**Methods:**

MR imaging examination was performed with a GE Signa EXCITE 3.0 T MRI scanner. The optimal b value in DWI with a learning cohort of patients was determined. The diagnostic accuracy for active lesions and disease activity were accessed by MRE combined with DWI.

**Results:**

The b value 800 s/mm^2^ group showed the highest diagnostic sensitivity (74.19%) for diagnostic assessment of active Crohn’s lesions on DWI. MRE combined with DWI showed the highest sensitivity (93.55%), specificity (89.47%) and diagnostic accuracy (92%) compared with MRE or DWI alone. The segmental MR score (MR-score-S) showed a significantly positive correlation with the Capsule Endoscopy Crohn’s Disease Activity Index Score (CECDAI-S) (*r* = 0.717, *p* < 0.01). The total MR score (MR-score-T) showed significant association with C-reactive protein (CRP) (*r* = 0.445, *p* = 0.019) and erythrocyte sedimentation rate (ESR) (*r* = 0.688, *p* < 0.01).

**Conclusions:**

MRE combined with DWI improves the diagnostic accuracy for active lesions and correlates the endoscopic disease activity. MRE with DWI could represent a non-invasive tool in assessing active inflammation in CD.

## Background

Crohn’s disease (CD) is characterized by chronic inflammation with a relapsing and remitting natural history. Treatment is generally effective in relieving clinical symptoms. However, preliminary evidence has suggested that therapeutic strategy for endoscopic remission might be associated with better outcome [1]. Mucosal healing (MH) or deep remission emerges as the therapeutic endpoint in patients with CD, especially in severe or complicated cases. Therefore, monitoring the disease with endoscopy and cross-sectional imaging is proposed to assess MH. Endoscopy allows direct visualization of the mucosa and acquisition of biopsies. In CD, although endoscopy is an invasive procedure and expensive in some countries, it is nearly the ‘golden’ standard for disease activity monitoring of luminal lesions. To determine the severity and therapeutic responses based on normalized, endoscopic manifestation, a variety of disease activity indices have been developed over decades [2,3]. For small bowel CD, the advent of capsule endoscopy has improved our understanding of the lining of the small bowel. Extensive consensus has established that capsule endoscopy visualize the whole small bowel, and it help to assess the small-bowel severity in CD [4]. However, capsule endoscopy also shows some limitations. The focal length of the vision is too short to measure the size of lesions reliably. However capsule endoscopy is not possible in stricturing disease, hence radiological imaging is usually required.

Cross-sectional imaging techniques as computed tomography enterography (CTE) and MRE may offer additional information on transmural inflammation, stricturing and fistulising complications [5]. CTE delineate the extent and severity of bowel wall inflammation, apart from detecting extra-luminal findings [6]. However, the increased spatial resolution of CT with new multidetectors carries along with the greater dose of ionizing radiation. In fact, effective dose of radiation for CTE is nearly five times higher than small bowel follow through [7]. MRE is preferred for the evaluation of the gastrointestinal tract because of the absence of ionizing radiation, along with the similar sensitivity and diagnostic accuracy of luminal and extraluminal lesions [8,9]. Most of the recent researches focused on the inflammatory activity of involved segments using gadolinium-enhanced T1-weighted sequence for evaluation of CD. MRE signs of inflammatory activity in the bowel segments showed a good correlation with the presence and severity of endoscopic lesions [10,11]. MRE can measure intestinal thickness, enhancement after contrast injection, edema and ulceration or even more sophisticated measure as diffusion weighted imaging (DWI). DWI derives its image contrast from differences in the motion of water molecules between tissues [12]. DWI has been reported for colonic lesions in active CD, which could avoid the use of gadolinium injection [13]. Thus, the absence of gadolinium-induced nephrogenic systemic fibrosis indicates that DWI might be the proper method for long time follow-up in CD.

To quantify MRI assessment in each segment, Magnetic Resonance Index of Activity (MaRIA) is used and gets the satisfactory consistency with Crohn’s Disease Endoscopic Index of Severity (CDEIS). However, only few studies were found to evaluate small bowel inflammation using DWI to date. Therefore, the purpose of our study was to evaluate the diagnostic accuracy of MRI combined with DWI and MRE for determination of inflammation in small bowel CD.

## Methods

### Patients

From September 2010 to September 2012, a total of thirty-six consecutive active CD patients were included in our restrospective study, including nineteen males and seventeen females (Table 1). All patients underwent both MR examination and single/double balloon enteroscopy within the same week. All patients gave written informed consent to participate in the present study. The ethic approval was provided by the Institutional Review Board (IRB) of the Ren Ji Hospital, School of Medicine, Shanghai Jiao Tong University.

**Table 1:** **Characteristics of all patients**

	Mean ± deviation	Range (min, max)
Age (year)	27 ± 10	13, 63
Course of disease (month)	34 ± 30	1, 122
Leucocyte (10^9/L)	7.17 ± 2.66	2.72, 16.11
Erythrocyte (10^9/L)	4.32 ± 0.68	2.67, 5.58
Hemoglobin (g/L)	112.38 ± 20.67	60, 150
Platelet (10^9/L)	304.47 ± 97.02	103, 582
High sensitive CRP (mg/L)	22.20 ± 23.16	0.7, 98
Blood sedimentation (mm/h)	27.26 ± 19.22	1, 80
Albumin (g/L)	35.0 ± 5.20	26.2, 45.5

The criteria of enrollment included: Harvey-Bradshaw Crohn’s Disease Activity Index > 4; C-reactive protein (CRP) > 8 mg/L or erythrocyte sedimentation rate (ESR) > 20 mm/h; Ulcers in at least one segment of bowels. The criteria of exclusion were intolerance or contraindication to undergo single/double balloon enteroscopy or MRE; Pregnancy; Active infection.

### MRE protocol

MR imaging examination was performed with a GE Signa EXCITE 3.0 T MRI scanner (GE HealthCare, Milwaukee, WI, USA). Patients fasted for over 8 hours before examinations and took 2000 ml solution of Polyethylene Glycol Electrolyte (PGE) powder (Wanhe Inc., Shenzhen, China) for bowel preparation. Forty five minutes before scanning, additional 1000 ml PGE solution was administered orally to each patient for small bowel distention. Scopolamine 10 mg (Shanghai Xinyi Pharmaceuticals Co., Ltd., Shanghai, China) was administered intramuscularly 10 minutes before the examination.

After acquiring standard three-plane scout images with supine position, sequences were obtained from the abdomen and pelvis using an eight-channel, phased-array body coil. All sequences except T1W imaging were scanned with respiratory-triggering method. T2-weighted single shot fast spin-echo images (ssFSE) were acquired in axial and coronal plane. The scan parameters were as follows: TR/TE, 2000-3400/68 msec; slice thickness, 5 mm; interslice gap, 0 mm; matrix, 384*192; FOV, 42*25 cm; NEX, 0.6; SENSE factor, 2. Axial T1-weighted three-dimensional fast spoiled gradient echo (FSPGR) was acquired with breath-holding technique: TR/TE, 310/2.5 msec; slice thickness, 6 mm; interslice gap, 0 mm; matrix, 288*192; FOV, 35*25 cm; NEX, 0.5; SENSE factor, 2.

Scan parameters of DWI were as follows: TR/TE, 5820-6200/74-78 msec; slice thickness, 6 mm; interslice gap, 2 mm; matrix, 128*96; FOV, 40*28 cm; NEX, 4. The frequency direction was left to right. Diffusion-encoding gradients were applied as 5 b values from 0 to 2500 s/mm^2^ (0, 800, 1500, 2000 and 2500 s/mm^2^) along the three orthogonal directions of motion-probing gradients. The apparent diffusion coefficient (ADC) maps were automatically constructed on a pixel-by-pixel basis.

A 3D gradient echo T1 sequence (LAVA) was conducted before and after intravenous administration of Magnevist (0.1 ml/kg; Bayer Vital Gmbh, Germany) at a rate of 3 ml/s for dynamic study in coronal plane (arterial phase 30 s, portal phase 70 s and post-equilibrium phase 90 s after injection). TR/TE, 3.3/1.5 msec; slice thickness, 2.4 mm; interslice gap, −1.8 mm; matrix, 256*288; FOV, 40*40 cm; NEX, 0.7.

The acquisition time of the entire examination for each patient was approximately 30 minutes.

### MRI evaluation

#### Determine the optimal b value in DWI with a learning cohort of patients

##### Qualitative analysis

The manifestation of intestinal anatomic structure on DWI was evaluated on a three-point scale as follows: 0 = both inflammatory and normal intestinal wall structure was clear, 1 = inflammatory intestinal wall structure was clear while partial normal intestinal wall structure was vague, 2 = only partial positive or negative intestinal wall structure could be identified. The diagnostic efficacy of inflammatory lesions on DW images would be evaluated and compared with the results of enteroscopy.

##### Quantitative analysis

The signal-to-noise ratio (SNR) and contrast-to-noise ratio (CNR) in all four different b value groups would be measured. Image noise was measured from a large area (approximately 260 cm^2^) outside the abdomen parenchyma and defined as the standard deviation of background signal intensity. The signal contrast and ADCs would be measured on both normal and inflammatory bowel wall when these images were properly magnified, and ROI areas would be determined as maximum. On DW images, ROIs were placed on the segments where the signal was the most significant.

### MRE combined with DWI improves the diagnostic accuracy for active lesions

Three different protocols were adopted for image evaluation in this session. Protocol A: only DW imaging was used; protocol B: only (MRE) imaging was used, including T1WI, T2WI, and LAVA; protocol C: both DW imaging and MRE imaging were used. In protocol A, the intestinal segment would be evaluated as positive when it demonstrated significant high signal intensity on DWI and low signal intensity on ADC map. In protocol B, the segments would be evaluated as positive then it demonstrated high signal intensity on T2WI, wall thickening more than 3 mm and apparent enhancement in LAVA imaging. In protocol C, the segments would be evaluated as positive when it appeared abnormal on both DW and MRE imaging. The b value adopted here would depend on the outcome we gained in the part of “Determine the optimal b value in DWI with a learning cohort of patients”. The diagnostic effect of these three reading protocols would be compared with the results of enteroscopy.

### MRE combined with DWI correlates the endoscopic disease activity

Image evaluation was performed on the GE workstation (GE Healthcare, AW4.2). All images were evaluated by two experienced gastrointestinal radiologists (4 and 6 years of experience) who were blinded to clinical symptoms and results of enteroscopy. For any discrepancies in the data analysis, a joint reading session was performed to obtain consensus. In DW images, the intestinal segment would be evaluated as positive when it demonstrated significant high signal intensity on DWI and low signal intensity on ADC map. The b value adopted here would depend on the outcome we gained in the part of “Determine the optimal b value in DWI with a learning cohort of patients”. The small intestinal segments were defined with coronal T2WI sequence: duodenum, jejunum (left upper abdomen), proximal ileum (middle and left lower abdomen), distal ileum (right lower abdomen and pelvis) and terminal ileum (10 cm up to ileal valve). Based on a comprehensive review of the literature, eight radiological signs were studied: (1) DWI hyper-intensity, (2) layer differentiation of DWI hyper-intensity, (3) bowel wall enhancement after intravenous contrast medium administration, (4) differentiation between the mucosae-submucosa complex and the muscularis propria, (5) bowel wall thickening, (6) mesenteric edema, (7) the presence of ulcers, and (8) the presence of polypoid hyperplasia. Furthermore, the presence or absence of a radiological sign in a given segment was rated ‘1’ or ‘0’, respectively. A modified MR scoring system was adopted: The segmental MR score (MR-score-S) was calculated as the sum of the numerical values obtained for the eight radiological signs for a given segment. The total MR-score (MR-score-T) was calculated as the sum of the MR-score-S in individual patient.

### Endoscopic evaluation

The interval between MR examination and single/double balloon enteroscopy (SBE/DBE) was less than one weeks. Mean oral intubation depth was about 250 cm and Mean anal intubation depth was about 129 cm respectively. Biopsy specimens were taken from suspected lesions.

Endoscopic images were evaluated by two experienced gastroenterologists. Due to the lack of scoring system for enteroscopy, the severity and extent of lesions in small bowel were assessed by the Capsule Endoscopy Crohn’s Disease Activity Index (CECDAI) [14]. For individual patient, five intestinal segments were defined: duodenum, jejunum, proximal ileum, distal ileum and terminal ileum. The segmental CECDAI score (CECDAI-S) was calculated in each given segment, and the total CECDAI score (CECDAI-T) was defined as the sum of the CECDAI score in a patient.

### Statistical analysis

Statistical analysis was performed using SPSS 11.5. Quantitative variables are represented as the mean ± standard deviation, and discrete variables were represented as frequencies or percentages. The evaluation of CNR, SNR and ADCs weere performed using one-way ANOVA. The diagnostic accuracy was performed using Chi-Square comparison. The correlations between MR scores and endoscopic scores, as well as clinical and biological markers of disease activity were calculated by Spearmen rank correlation test.

## Results

### Characteristics of included subjects

In current study, a total of 100 intestinal segments were evaluated (36 terminal ileum segments, 33 distal ileum segments, 25 proximal ileum segments, 5 jejunal segments and 1 duodenal segment), which included 62 positive segments and 38 negative segments. Sixteen patients were diagnosed with complication: 3 cases of internal fistula, 5 cases of abdominal abscess, 4 cases of anal fistula and 4 cases of anal abscess.

### Determine the optimal b value in DWI with a learning cohort of patients

**Figure 1:**
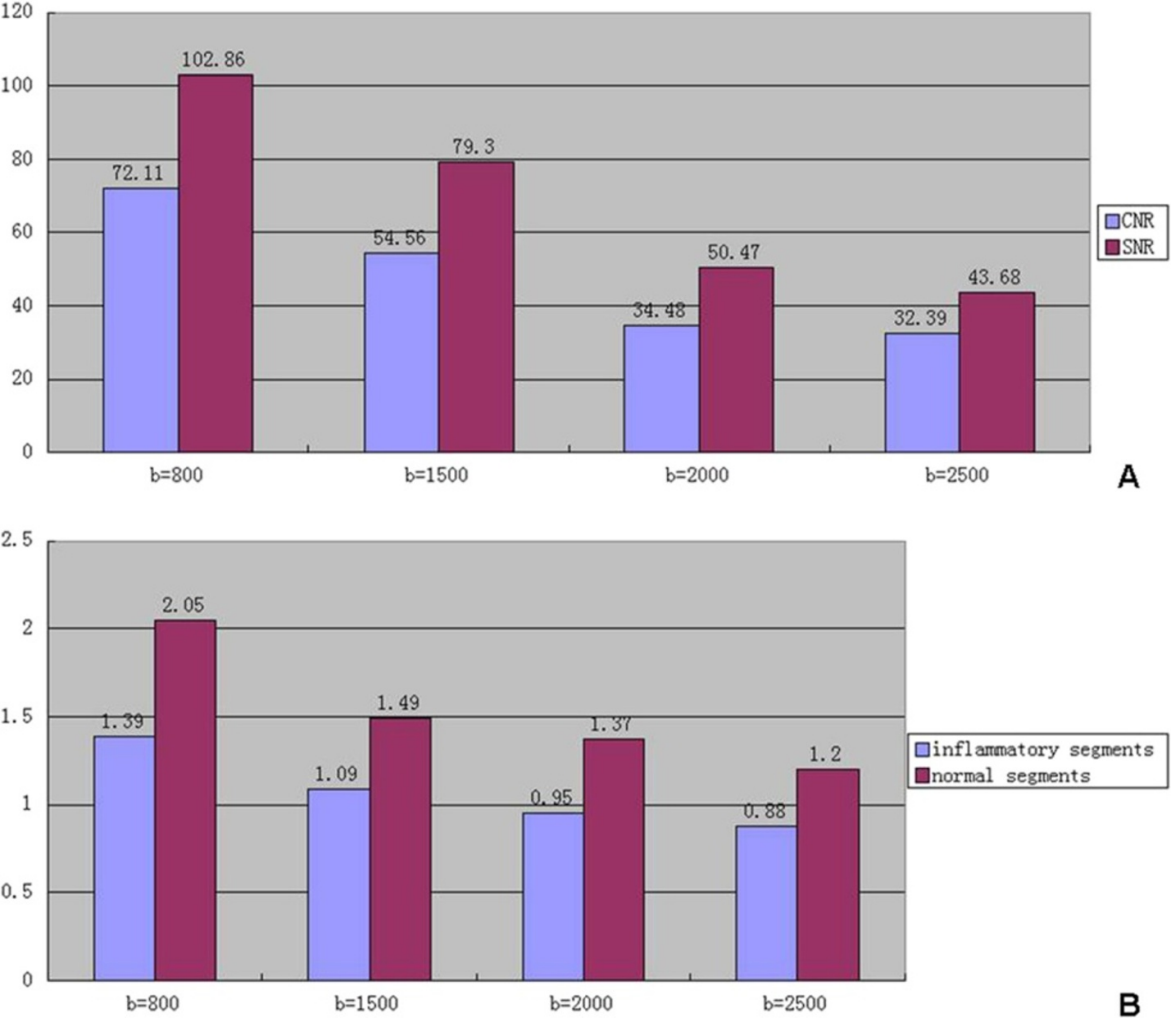
**Determine the optimal b value in DWI with a learning cohort of patients.** The mean CNR and SNR of four b value sequences. The unit of b value is s/mm^2^**(A)**. The ADC values of four b value sequences. The unit of ADC is 10^−3^ mm^2^/s **(B)**.

SNR and CNR decreased remarkably when b value 2000 s/mm^2^ and 2500 s/mm^2^ was adopted. There was significant differences existed in SNR (*F* = 17.074, *p* < 0.01) and CNR (*F* = 14.920, *p* < 0.01) when b value as 800 s/mm^2^ and 1500 s/mm^2^, respectively. The ADCs of the inflammatory intestinal segments were significantly lower compared with those in the normal segments using all four b values (*p* < 0.01) (Figure 1). It was indicated that no matter what b value was chosen, the ADC difference between inflammatory and normal bowel segments was significant, and it was hard to tell which b value was better.

**Figure 2:**
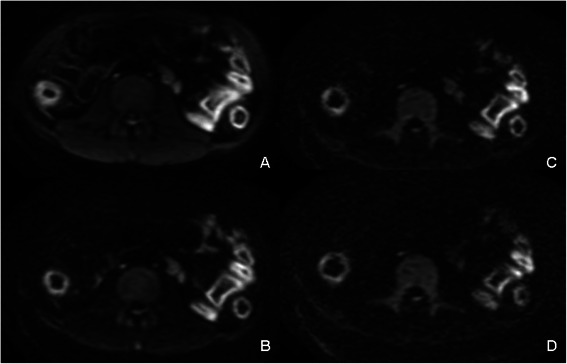
**Qualitative image quality comparison among four b values.** DWI images were obtained from a 30-year-old man. The Inflammatory lesion of approximal ileum was detected by endoscopy. When b = 800 s/mm^2^ was adopted, the lesion was demonstrated clearly as well as normal small intestinal wall, enlarged lymph nodes and abdominal wall structure **(A)**. When b = 1500 s/mm^2^ was adopted, normal small intestinal wall could barely be identified **(B)** and when b = 2000 s/mm^2^ or b = 2500 s/mm^2^ was adopted **(C and D)**, even abdominal wall structure became vague. The enlarged lymph nodes could not be identified when b = 2500 s/mm^2^ was adopted **(D)**.

Considering the optimal image quality of DWI, the b value 800 s/mm^2^ was adopted as scale 0. When b value 1500 s/mm^2^ was selected, 7 cases were evaluated as scale 1 and 29 cases as scale 0. When b value 2000 s/mm^2^ was selected, 11 cases were evaluated as scale 1 and the other 25 cases were evaluated as scale 2. When b value 2500 s/mm^2^ was selected, all cases were evaluated as scale 2 (Figure 2).

**Figure 3:**
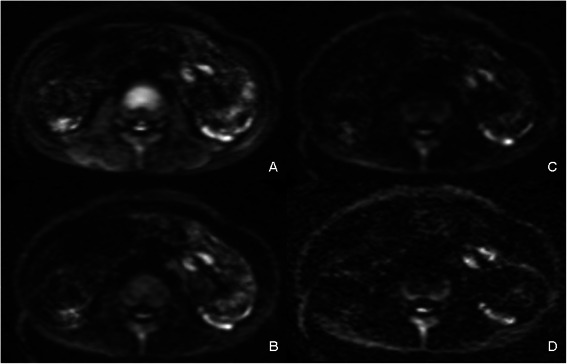
**Diagnostic effect of different b value groups.** In a 28-year-old man, high signal intensity in approximal ileum could be detected when b value was 800 s/mm^2^**(A)** or 1500 s/mm^2^**(B)** was chosen. However, when b value was 2000 s/mm^2^ or 2500 s/mm^2^**(C and D)** was selected, the high signal was apparently depressed and the lesion was missed.

The b value 800 s/mm^2^ group showed the highest diagnostic sensitivity (74.19%) for diagnostic assessment of active Crohn’s lesions on DWI. However, the specificity was quite low in b value 800 s/mm^2^ group (39.47%). Among four different b groups, the diagnostic accuracy was similar (all *p* > 0.05) (Figure 3).

### MRE combined with DWI improves the diagnostic accuracy for active lesions

**Figure 4:**
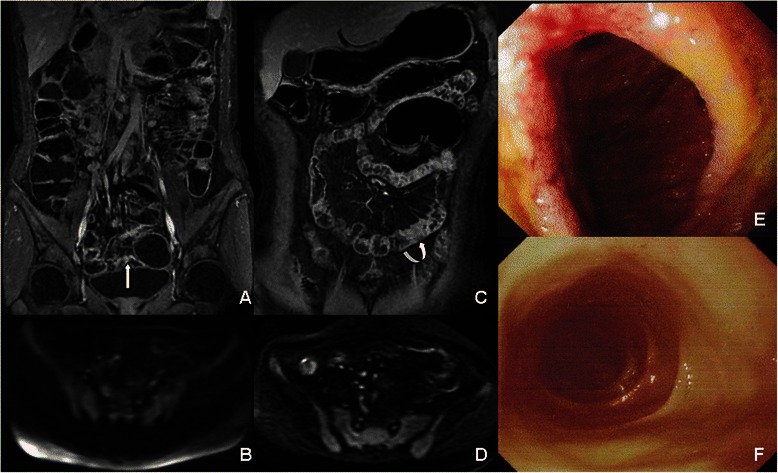
**The combination of MRE and DWI could improve diagnostic sensitivity and specificity.** A 34-year-old female patient suffered from active CD with multi-segmental lesions in ileum **(A, B and E)**. On LAVA image **(A)** abnormal enhancement could be detected in pelvis (white arrow), which was confirmed by enteroscopy **(E)**, While on DWI **(B)**, no abnormal high signal intensity lesion was found in the same cross-sectional slice. A 42-year-old male patient suffered from active CD **(C, D and F)**. On LAVA image, suspicious local bowel wall thickening (curved arrow-head) was detected in distal ileum **(C)**, while on DWI **(D)**, no high signal intensity was found in the same cross-sectional slice, which was confirmed by enteroscopy **(F)**.

**Figure 5:**
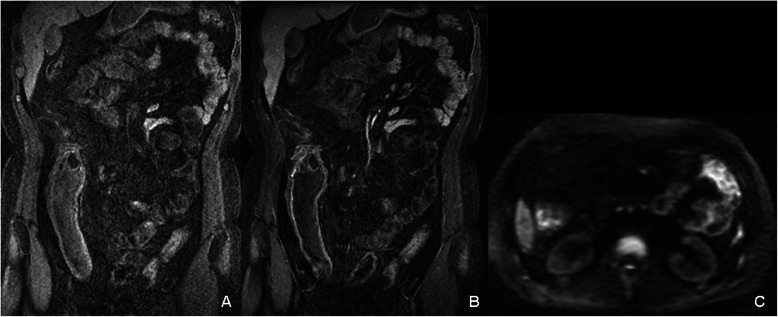
**The combination of MRE and DWI could improve diagnostic sensitivity and specificity.** A thirty-three male patient suffered from active CD. On LAVA image the jejunum (right upper abdomen) was not distended very well and it was difficult to decide whether it had inflammatory lesion because of high signal intensity both before **(A)** and after **(B)** contrast injection. While on DWI **(C)**, abnormal high signal intensity was found in the same cross-sectional slice indicating the inflammatory lesion, which was confirmed by enteroscopy.

The diagnostic accuracy of inflammatory segments using three reading protocols was also assessed. MRE combined with DWI showed the highest sensitivity (93.55%), specificity (89.47%) and diagnostic accuracy (92%) compared with MRE or DWI alone (Table 2). The combination of MRE and DWI could improve diagnostic sensitivity and specificity (Figures 4 and 5).

**Table 2:** **Diagnostic assessment of active Crohn’s lesions**

Reading protocol	TP	FP	FN	TN	Sensitivity	Specificity	Diagnostic accuracy
A	42	14	20	24	74.19%	63.16%	66%
B	51	10	11	28	82.26%	73.68%	79%
C	58	4	4	34	93.55%	89.47%	92%

### MRE combined with DWI correlates the endoscopic disease activity

**Figure 6:**
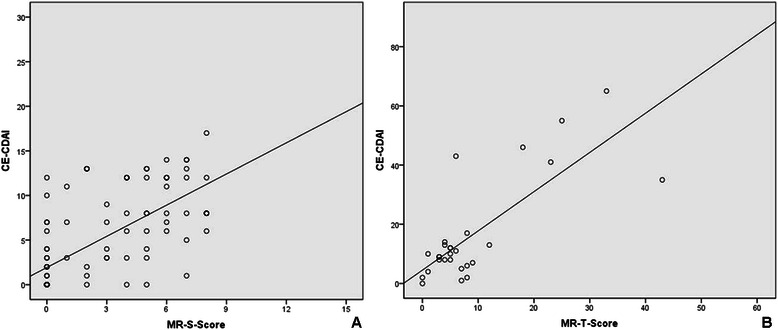
**The correlation between MR score and CECDAI score.** Both MR-score-S and MR-score-T showed a significantly positive correlation with CECDAI-S (*r* = 0.717, *p* < 0.01) and CECDAI-T (*r* = 0.535, *p* < 0.01).

In segmental analysis, MR-score-S showed a significantly positive correlation with CECDAI-S (*r* = 0.717, *p* < 0.01). Similarly, MR-score-T was found to be significantly correlated with CECDAI-T (*r* = 0.535, *p* < 0.01) in individual patient (Figure 6)

.

**Figure 7:**
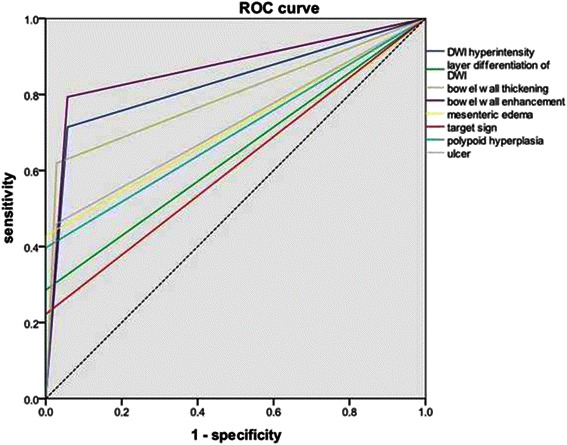
**The ROC curve of predicting CD activity.** The ROC curve indicated that bowel wall enhancement was the optimal predictor of inflammation (AUROC = 0.87). Besides, DWI hyper-intensity (AUROC = 0.83) and bowel wall thickening (AUROC = 0.80) could also predict active inflammation pretty well. At the same time, the layer differentiation of DWI hyper-intensity and differentiation between the mucosaesubmucosa complex and the muscularis propria presented the perfect diagnostic specificity.

Moreover, to determine the specific signs predicting clinical and endoscopic disease activity, Receiver Operator Curves (ROC) were used. The Area Under the Receiver Operator Curve (AUROC) indicated that bowel wall enhancement was the optimal predictor of inflammation (AUROC = 0.87) with the satisfactory sensitivity (79.4%) and specificity (94.3%). Besides, DWI hyper-intensity (AUROC = 0.83) and bowel wall thickening (AUROC = 0.80) could also predict active inflammation pretty well. Interestingly, It was demonstrated that the layer differentiation of DWI hyper-intensity and differentiation between the mucosaesubmucosa complex and the muscularis propria presented the perfect diagnostic specificity (Figure 7).

Then, we focused on the correlation between MR findings and clinical disease activity index or biomarkers. Although MR-score-T showed significant association with CRP (*r* = 0.445, *p* = 0.019) and ESR (*r* = 0.688, *p* < 0.01), we could not find significant association between MR-score-T and CDAI (*r* = 0.299, *p* = 0.146).

## Discussion

CD is a chronic inflammatory disorder which may affect any part of the gastrointestinal tract, frequently involving the small and large bowel. It is difficult to completely observe the whole small bowel with conventional endoscopy and MRI is now increasingly used. DWI has the capability to detect inflammatory lesion and its utility in CD activity has been assessed in several studies, while few papers ever discussed the reason of b value selection. Oussalah *et al.* [13] reported the dynamic contrast-enhanced MRI (DCE-MRI) and DWI with *b* values 0 and 600 s/mm^2^. It was also reported that three *b* values (0, 100 and 800 s/mm^2^) could be used with axial images through the upper and lower abdomen obtained in Crohn’s disease [15]. Neubauer *et al.* [16] reported the utility of DWI in children and young adults suffering from Crohn’s disease with *b* values 50 and 800 s/mm^2^. In our study, we compared four different b values and it turned out that 800 s/mm^2^ was the best choice in this MRE-DWI examination. Not only the image with *b* value 800 s/mm^2^ had the best SNR and CNR, but it showed the highest diagnostic sensitivity (74.19%) and the diagnostic accuracy of it was similar to others.

SBE and DBE can provide high-quality images of the small intestinal mucosa, which enables the gastroenterologists, receive integral impression and evaluation of involved small intestine immediately. Due to the lack of scoring system for enteroscopy, the severity and extent of lesions in small bowel were assessed by the CECDAI in the present study. CECDAI can diagnose CD, exclude normal mucosal breaks, identify damage induced by nonsteroidal anti-inflammatory drugs, measure disease activity and severity, assess therapeutic response for clinical trials, and determine medical management for the patient with small-bowel CD. However, CECDAI is not suitable for patients with suspicious stenosis or obstruction due to the contraindication of capsule endoscopy (CE).

Some studies had proposed to evaluate disease activity with scoring systems based on combination of MRI features [13,17]. MRE is noninvasive, and the intestinal wall adjacent to the obstruction could be clearly displayed. In CD, the MR-score-T has been indicated to correlate with the endoscopic disease activity, even in colonography without bowel preparation [13]. In the present study, we found the similar evidence that both MR-score-S and MR-score-T were correlated with CECDAI. And bowel wall enhancement shows the best predictive value for active inflammation in our study. The level of bowel wall enhancement after intravenous administration of gadolinium is the single, most reliable MRI criterion for disease activity because that it reflects wall vascularity and vessel permeability, which increased in active inflammation [18,19]. Indeed, several studies have proved bowel wall thickening as an independent predictor of inflammation in CD [13,20]. Interestingly, some MR features as the wall thickening showed the lower value to assess inflammatory activity in a few reports [21]. We noticed that without proper bowel preparation and luminal distension, the wall thickening caused by constriction sometimes could be easily confused with inflammatory lesion even after contrast injection. In fact, normal signal intensity of thickened wall could appear in chronic inflammation or even normal bowel wall without properly distention [22]. Sometimes hyper-enhancement of the mucosa may be the only manifestation of active inflammation without any significant bowel thickening, which can be found in patients with recurrent CD after fibrotic reaction [23].

In our study, DWI hyper-intensity predicted inflammatory activity pretty well, which was closed to bowel wall enhancement. Fortunately, DWI hyper-intensity has been found similar possibility on predicting disease active in colon [13]. The layer differentiation of DWI hyper-intensity could be observed in all cases with active inflammation. MR feature of differentiation between the mucosaesubmucosa complex and the muscularis propria also showed the similar pattern in our study. The specific mechanism is still to be determined. The inner layer with higher signal might indicate granulomatous or fibrotic reaction, and the outer layer with lower signal might indicate bowel wall edema based on pathologic findings of CD.

The MaRIA score was recently prospectively validated and subsequently considered as the benchmark for assessing inflammatory activity in MRI for ileocolonic CD [10,24]. Wall thickening, relative contrast enhancement, edema and ulcer were the main parameters while no DWI factor was included. Rimola J *et al*. suggested that the creation of MaRIA-DWI score could be the better activity index for small bowel CD [25]. In this score system, ADC value was added as a new parameter. Unfortunately, the data they got was not compared with gold standard and the ADC value itself could be variable according to different b value or MR scanner. So we adopt the scoring system using by Oussalah *et al*. and added other radiological signs in it which we named “modified MR scoring system”, which we expected could be more stable and dependable.

Although the MR-score-T was correlated with both ESR and CRP, it did not show significant correlation with clinical disease activity index in CD. The CDAI score reflects patients’ general condition including intestinal and parenteral manifestations. It’s a relatively subjective evaluation score depending much more on patients’ chief complain than clinical, endoscopic or radiologic test, which might cause the bias between MR-score-T and CDAI score. Furthermore, it should be noted that MRI could not replace endoscopy in evaluating disease activity because of its difficulty in assessing mucosal status. However, MRI could be adapted as a useful technique for long-time follow-up.

There were several limitations to this study. First, the correlation between bowel wall ADCs and other MRI markers of disease activity were not well investigated at present. Although ADC of DWI has preliminarily been demonstrated as an radiological, disease activity marker in CD [26,27], sometimes endoscopic finding could not confirm the ADCs findings in the same segments of inflammatory bowel. Second, in CD, both inflammation and fibrosis usually coexisted in the same bowel segments [28,29]. However, it’s still very difficult to identify whether thickened bowel wall contains substantial fibrosis with these imaging sequences.

## Conclusions

In conclusion, when DWI was combined with MRE for CD activity evaluation, b value 800 s/mm^2^ was recommended as the optimal DWI scanning parameter because of its good image quality and high diagnostic sensitivity of lesion detection. When diagnostic accuracy of active lesion was concerned, it was concluded that the combination of DWI and MRE showed the highest sensitivity, specificity and diagnostic accuracy compared with MRE or DWI alone. At the same time, the MR score obtained from both DWI and MRE radiological characteristics had showed a significantly positive correlation with CECDAI. Among all the MR score signs, bowel wall enhancement was the optimal predictor of active inflammation.

## References

[1] Papi C, Fasci-Spurio F, Rogai F, Settesoldi A, Margagnoni G, Annese V (2013). Mucosal healing in inflammatory bowel disease: treatment efficacy and predictive factors. Digestive Liver Di Off J Italian Soc Gastroenterol Italian Assoc Study Liver.

[2] Sandborn WJ, Feagan BG, Hanauer SB, Lochs H, Lofberg R, Modigliani R (2002). A review of activity indices and efficacy endpoints for clinical trials of medical therapy in adults with Crohn’s disease. Gastroenterology.

[3] Daperno M, D’Haens G, Van Assche G, Baert F, Bulois P, Maunoury V (2004). Development and validation of a new, simplified endoscopic activity score for Crohn’s disease: the SES-CD. Gastrointest Endosc.

[4] Bourreille A, Ignjatovic A, Aabakken L, Loftus EV, Eliakim R, Pennazio M (2009). Role of small-bowel endoscopy in the management of patients with inflammatory bowel disease: an international OMED-ECCO consensus. Endoscopy.

[5] Panes J, Bouzas R, Chaparro M, Garcia-Sanchez V, Gisbert JP, de GB M (2011). Systematic review: the use of ultrasonography, computed tomography and magnetic resonance imaging for the diagnosis, assessment of activity and abdominal complications of Crohn’s disease. Aliment Pharmacol Ther.

[6] Huprich JE, Fletcher JG (2009). CT enterography: principles, technique and utility in Crohn’s disease. Eur J Radiol.

[7] Jaffe TA, Gaca AM, Delaney S, Yoshizumi TT, Toncheva G, Nguyen G (2007). Radiation doses from small-bowel follow-through and abdominopelvic MDCT in Crohn’s disease. AJR Am J Roentgenol.

[8] Giusti S, Faggioni L, Neri E, Fruzzetti E, Nardini L, Marchi S (2010). Dynamic MRI of the small bowel: usefulness of quantitative contrast-enhancement parameters and time-signal intensity curves for differentiating between active and inactive Crohn’s disease. Abdom Imaging.

[9] Bernstein CN, Greenberg H, Boult I, Chubey S, Leblanc C, Ryner L (2005). A prospective comparison study of MRI versus small bowel follow-through in recurrent Crohn’s disease. Am J Gastroenterol.

[10] Rimola J, Ordas I, Rodriguez S, Garcia-Bosch O, Aceituno M, Llach J (2011). Magnetic resonance imaging for evaluation of Crohn’s disease: validation of parameters of severity and quantitative index of activity. Inflamm Bowel Dis.

[11] Steward MJ, Punwani S, Proctor I, Adjei-Gyamfi Y, Chatterjee F, Bloom S (2012). Non-perforating small bowel Crohn’s disease assessed by MRI enterography: derivation and histopathological validation of an MR-based activity index. Eur J Radiol.

[12] Laurent V, Trausch G, Bruot O, Olivier P, Felblinger J, Regent D (2010). Comparative study of two whole-body imaging techniques in the case of melanoma metastases: advantages of multi-contrast MRI examination including a diffusion-weighted sequence in comparison with PET-CT. Eur J Radiol.

[13] Oussalah A, Laurent V, Bruot O, Bressenot A, Bigard MA, Regent D (2010). Diffusion-weighted magnetic resonance without bowel preparation for detecting colonic inflammation in inflammatory bowel disease. Gut.

[14] Gurudu SR, Leighton JA (2009). Assessment and validation of the new capsule endoscopy Crohn’s disease activity index (CECDAI): what difference does it make?. Inflamm Bowel Dis.

[15] Griffin N, Grant LA, Anderson S, Irving P, Sanderson J (2012). Small bowel MR enterography: problem solving in Crohn’s disease. Insights Imaging.

[16] Neubauer H, Pabst T, Dick A, Machann W, Evangelista L, Wirth C (2013). Small-bowel MRI in children and young adults with Crohn disease: retrospective head-to-head comparison of contrast-enhanced and diffusion-weighted MRI. Pediatr Radiol.

[17] Ajaj WM, Lauenstein TC, Pelster G, Gerken G, Ruehm SG, Debatin JF (2005). Magnetic resonance colonography for the detection of inflammatory diseases of the large bowel: quantifying the inflammatory activity. Gut.

[18] Maccioni F, Bruni A, Viscido A, Colaiacomo MC, Cocco A, Montesani C (2006). MR imaging in patients with Crohn disease: value of T2- versus T1-weighted gadolinium-enhanced MR sequences with use of an oral superparamagnetic contrast agent. Radiology.

[19] Fidler JL, Guimaraes L, Einstein DM (2009). MR imaging of the small bowel. Radiographics Rev Publ Radiol Soc North Am Inc.

[20] Martinez MJ, Ripolles T, Paredes JM, Blanc E, Marti-Bonmati L (2009). Assessment of the extension and the inflammatory activity in Crohn’s disease: comparison of ultrasound and MRI. Abdom Imaging.

[21] Low RN, Sebrechts CP, Politoske DA, Bennett MT, Flores S, Snyder RJ (2002). Crohn disease with endoscopic correlation: single-shot fast spin-echo and gadolinium-enhanced fat-suppressed spoiled gradient-echo MR imaging. Radiology.

[22] Udayasankar UK, Martin D, Lauenstein T, Rutherford R, Galloway J, Tudorascu D (2008). Role of spectral presaturation attenuated inversion-recovery fat-suppressed T2-weighted MR imaging in active inflammatory bowel disease. J Magnetic Resonance Imaging JMRI.

[23] Sinha R, Verma R, Verma S, Rajesh A (2011). MR enterography of Crohn disease: part 2, imaging and pathologic findings. AJR Am J Roentgenol.

[24] Rimola J, Rodriguez S, Garcia-Bosch O, Ordas I, Ayala E, Aceituno M (2009). Magnetic resonance for assessment of disease activity and severity in ileocolonic Crohn’s disease. Gut.

[25] Buisson A, Joubert A, Montoriol PF, Da Ines D, Hordonneau C, Pereira B (2013). Diffusion-weighted magnetic resonance imaging for detecting and assessing ileal inflammation in Crohn’s disease. Aliment Pharmacol Ther.

[26] Kiryu S, Dodanuki K, Takao H, Watanabe M, Inoue Y, Takazoe M (2009). Free-breathing diffusion-weighted imaging for the assessment of inflammatory activity in Crohn’s disease. J Magnetic Resonance Imaging JMRI.

[27] Ream JM, Dillman JR, Adler J, Khalatbari S, McHugh JB, Strouse PJ (2013). MRI diffusion-weighted imaging (DWI) in pediatric small bowel Crohn disease: correlation with MRI findings of active bowel wall inflammation. Pediatr Radiol.

[28] Adler J, Swanson SD, Schmiedlin-Ren P, Higgins PD, Golembeski CP, Polydorides AD (2011). Magnetization transfer helps detect intestinal fibrosis in an animal model of Crohn disease. Radiology.

[29] Adler J, Punglia DR, Dillman JR, Polydorides AD, Dave M, Al-Hawary MM (2012). Computed tomography enterography findings correlate with tissue inflammation, not fibrosis in resected small bowel Crohn’s disease. Inflamm Bowel Dis.

